# Targeted therapies in CLL/SLL and the cumulative incidence of infection: A systematic review and meta-analysis

**DOI:** 10.3389/fphar.2022.989830

**Published:** 2022-09-14

**Authors:** Stephanos Vassilopoulos, Fadi Shehadeh, Markos Kalligeros, Quynh-Lam Tran, Fred Schiffman, Eleftherios Mylonakis

**Affiliations:** ^1^ Infectious Diseases Division, Rhode Island Hospital, Providence, RI, United States; ^2^ Warren Alpert Medical School of Brown University, Providence, RI, United States; ^3^ School of Electrical and Computer Engineering, National Technical University of Athens, Athens, Greece; ^4^ Division of Hematology-Oncology, Rhode Island Hospital and The Miriam Hospital, Providence, RI, United States

**Keywords:** chronic lymphocytic leukemia (CLL), infections, targeted therapies, ibrutinib, idelalisib, monoclonal antibodies

## Abstract

**Background:** Patients with chronic lymphocytic leukemia (CLL)/small lymphocytic lymphoma (SLL) are prone to infections.

**Aims:** Provide a pooled estimate of the cumulative incidence for infections that fulfilled the criteria associated with severe infectious adverse events for grade 3 or higher (including pneumonia, febrile neutropenia and sepsis) in patients who receive targeted therapies.

**Methods:** We searched PubMed and EMBASE for randomized controlled trials (RCT) that included patients with CLL/SLL who received targeted therapies and performed a random-effects meta-analysis to estimate the cumulative incidence of infections.

**Results:** Of 2,914 studies screened, we retrieved 31 which evaluated 11,660 patients. The pooled cumulative incidence of infections for patients who received treatment regimens based on a BTK inhibitors was 19.86%. For patients who received treatment based on rituximab and second generation anti-CD20 monoclonal antibodies, the pooled cumulative incidence of infections was 19.85 and 13.46%, respectively. Regarding PI3K inhibitor-based regimens the cumulative incidence of severe infections was 30.89%. BCL-2 inhibitors had a cumulative incidence of infections of 17.49% while lenalidomide and alemtuzumab had an incidence of 13.33 and 45.09%, respectively. The cumulative incidence of pneumonia ranged from 3.01 to 8.45% while febrile neutropenia ranged from 2.68 to 10.80%. Regarding sepsis, the cumulative incidence ranged from 0.9 to 4.48%.

**Conclusion:** Patients with CLL/SLL who receive targeted therapies may develop severe infections at significant rates that, in addition to disease stage and other complications, depend on the mechanism of action of the used drug. Surveillance for infections and development of effective prophylactic strategies are critical for patients with CLL/SLL who receive targeted therapies.

**Systematic Review Registration:** [https://systematicreview.gov/], identifier [registration number]

## 1 Introduction

Chronic lymphocytic leukemia (CLL), and its nodal form small lymphocytic lymphoma (SLL), is the most common leukemia in Western countries with a median age of 70 years at diagnosis (Smith et al., 2011; Swerdlow et al., 2016). Patients with CLL/SLL are at increased risk for infections and almost 60% of deaths in this patient population are attributed to infections (Morrison, 2014). The predisposition of patients with CLL/SLL to infections is associated with disease processes and utilization of various treatments (Hensel et al., 2003; Wadhwa and Morrison, 2006; Hilal et al., 2018). Hypogammaglobulinemia (with IgG3 and IgG4 being most affected), cell-mediated immunity defects, complement deficiencies and alterations on neutrophil/monocyte count and activity make CLL/SLL patients more susceptible to infections (Itälä et al., 1996; Scrivener et al., 2003; Gonzalez-Rodriguez et al., 2010; Forconi and Moss, 2015; Parikh et al., 2015).

Several studies have demonstrated development of severe infections in patients that are being treated for CLL/SLL, both with backbone alkylating agents/purine analogs and with targeted treatments (Anaissie et al., 1998; Hensel et al., 2003; Teh et al., 2018). The use of established alkylating agents (e.g., chlorambucil) has been associated with an increased incidence of bacterial infections, specifically *Staphylococcus aureus*, *Streptococcus pneumoniae*, *Haemophilus influenzae*, *Klebsiella pneumoniae*, and *Escherichia coli* (Morrison, 2009; Morrison, 2014). Markedly, purine analogs (specifically fludarabine), have been linked with opportunistic infections such as *Listeria monocytogenes*, *Mycobacterium* spp, *Pneumocystis jirovecii* and herpes viruses (Anaissie et al., 1998; Morrison, 2014).

Treatment of CLL/SLL has entered an era that notably focuses on the utilization of targeted therapies such as Bruton tyrosine kinase (BTK) inhibitors (BTKi), drugs that target B-cell lymphoma-2 protein (BCL-2 inhibitors), anti-CD20 monoclonal antibodies, and phosphoinositide 3-kinase (PI3K) inhibitors. These targeted treatments have improved the prognosis of patients with CLL/SLL by providing individualized approaches and favorable safety profiles compared with backbone chemoimmunotherapy (Burger and O’Brien, 2018; Yosifov et al., 2019). However, increased use of such drugs can also predispose to infection development (Hilal et al., 2018). Taking into consideration the rising use of targeted agents, we performed a systematic-review and meta-analysis of randomized clinical trials to estimate the incidence of infections, in patients with CLL/SLL who receive them.

## 2 Materials and methods

### 2.1 Data sources and search strategy

For this meta-analysis we followed the Preferred Reporting Items for Systematic Reviews and Meta-Analyses Statement (PRISMA) statement checklist (Page et al., 2021). We searched PubMed/MEDLINE and EMBASE databases for literature in English, using the following search term (“chronic lymphocytic leukemia” OR “small lymphocytic lymphoma” OR CLL OR SLL) AND (randomized OR randomly). We also manually searched the reference lists for additional eligible studies.

### 2.2 Study selection

We included randomized controlled trials that fulfilled the following criteria: a) randomized patients with CLL/SLL, b) used drug regimens that included BTKi, anti-CD20 monoclonal antibodies, PI3K inhibitors, BCL-2 inhibitors, anti-CD52 monoclonal antibody (alemtuzumab), or lenalidomide, c) compared the effect of a CLL/SLL treatment with a control (different regimen or placebo), and d) included extractable safety data of ≥1 outcomes of interest. Lenalidomide is approved by the US Food and Drug Administration for treatment of multiple myeloma and 5q-myelodysplastic syndrome and has a promising clinical activity in CLL/SLL for which it is under evaluation, so we included it in this analysis.

We focused on patients who received treatment regimens against their disease and not on the complications of a hematopoietic stem cell transplantation and excluded studies that involved hematopoietic stem cell transplantation. For studies with multiple extended follow-up reports, we included the results of the first published reports, as these included more data on infectious complications. We excluded review articles, case reports, meeting abstracts and reports from non-randomized, single-arm trials, case-control, cross-sectional and observational studies. Two reviewers (SV and QLT) screened titles and abstracts independently to evaluate eligibility and performed full text screening of selected studies. Any discrepancies were resolved by discussion.

### 2.3 Primary and secondary outcomes

We retrieved data regarding the grade of infectious complications based on the National Cancer Institute Common Terminology Criteria for Adverse Events (CTCAE) and assessed the incidence of severe infections that occurred during the follow-up period (National Cancer Institute, 2021). We used the term “cumulative incidence” to refer to the infection rate measured during the follow-up period of retrieved RCTs (Munn et al., 2015). For primary outcome we evaluated the incidence of infections that fulfilled the criteria associated with grade 3 or higher adverse events and for secondary outcomes the incidence of pneumonia grade 3 or higher on the scale of adverse events, febrile neutropenia, and sepsis. Grade 3 infectious adverse events are severe or medically significant infections that require hospitalization but are not life-threatening. Grade 4 infectious adverse events endanger patient lives and require urgent intervention, while grade 5 infectious adverse events are the cause of death of a patient. Throughout this meta-analysis grade 3 or higher infectious adverse events are characterized as severe infectious events.

### 2.4 Data extraction and quality assessment

Extracted data included randomized safety populations, intervention regimens, outcomes of interest, and quality information. We retrieved the number of patients who developed infections, as studies reported their results in that way, and not the number of infectious events. We evaluated the risk of bias using the revised Cochrane risk-of-bias tool for randomized trials (RoB 2), which assesses the validity and bias in randomized trials across five domains: randomization process, deviations from intended interventions, missing outcome data, measurement of the outcome, and selection of the reported result (Sterne et al., 2019).

### 2.5 Data synthesis and analysis

For data analysis we used the Stata v17 Software (Stata Corporation, College Station, TX). Studies and regimens were grouped according to mechanism of action. The treatments that we included in our analysis had to be reported in two or more different studies. We used the DerSimonian and Laird approach (DerSimonian and Laird, 1986) to perform a random-effects meta-analysis and estimate the cumulative incidence of severe infections among patients receiving targeted CLL/SLL treatment regimens. We utilized a random-effects approach as we assumed that the effects are heterogeneous due to differences in study design, drug dosages and combination of agents used by each study. To stabilize the variances, we used the Freeman Tukey double arcsine transformation (Nyaga et al., 2014).

We sub-grouped the studies by treatment setting (treatment naïve versus relapsed/refractory). For randomized controlled trials (RCTs) with BTKi-based regimens we sub-grouped them to those that used monotherapy and those that used combination treatment. Similarly, studies that used second generation anti-CD20 monoclonal antibody-based regimens were sub-grouped by monotherapy versus combination therapy. For the analysis of secondary outcomes, we followed the same approach. Regarding the heterogeneity estimation we used the I^2^ statistic (Higgins and Thompson, 2002) and to explore publication bias and small study effects we used the Egger’s test (Peters et al., 2006). For this meta-analysis and systematic review, we utilized confidence intervals of 95%.

## 3 Results

**FIGURE 1:**
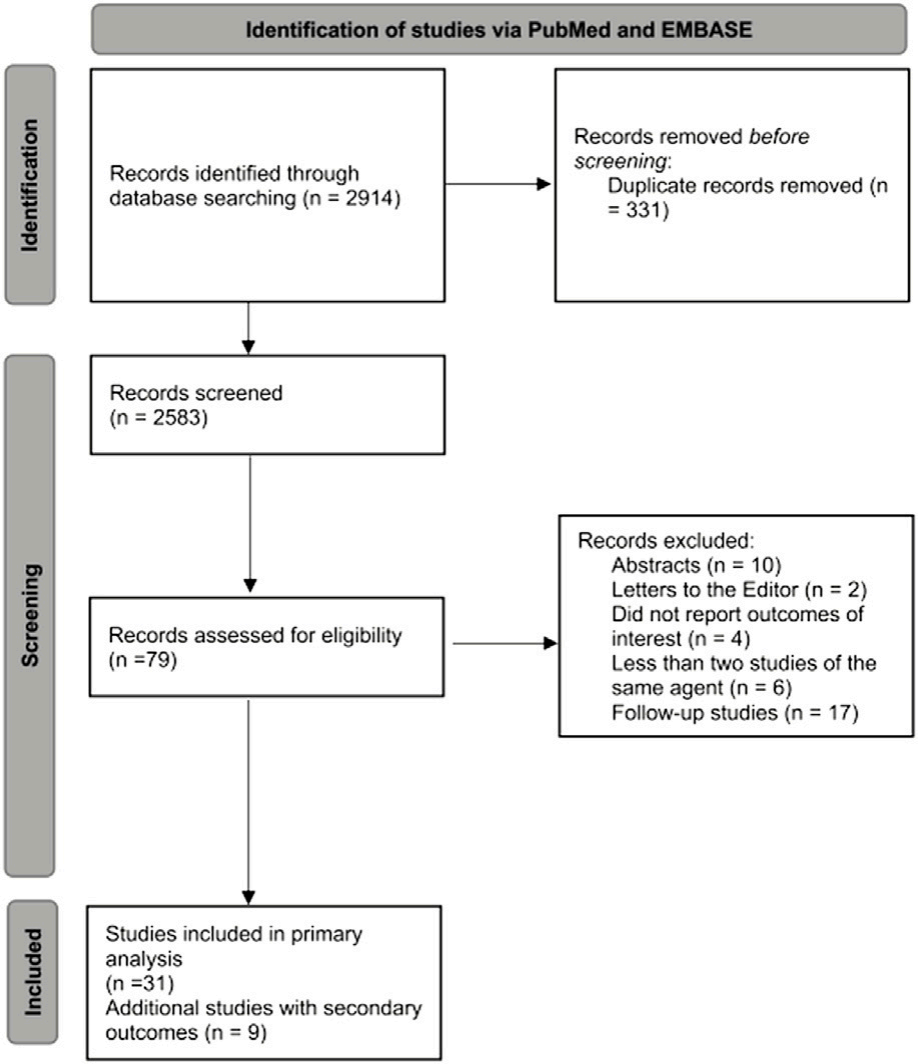
Flow diagram for selection of studies included in the systematic review and meta-analysis.

In total we retrieved 2,914 citations from the PubMed and EMBASE searches. After title and abstract screening, we excluded 2,583 studies and reviewed 79 publications in detail. Overall, we identified 31 clinical trials that included extractable data for our primary outcome (Wendtner et al., 2004; Hallek et al., 2010; Robak et al., 2010; Elter et al., 2011; Byrd et al., 2014; Furman et al., 2014; Geisler et al., 2014; Goede et al., 2014; Hillmen et al., 2015; van Oers et al., 2015; Chanan-Khan et al., 2016; Eichhorst et al., 2016; Greil et al., 2016; Chanan-Khan et al., 2017; Fink et al., 2017; Jones et al., 2017; Robak et al., 2017; Zelenetz et al., 2017; Dartigeas et al., 2018; Michallet et al., 2018; Seymour et al., 2018; Woyach et al., 2018; Burger et al., 2019; Fischer et al., 2019; Shanafelt et al., 2019; Ghia et al., 2020; Sharman et al., 2020; Byrd et al., 2021; Jindal et al., 2021; Wierda et al., 2021; Langerbeins et al., 2022). We additionally found nine studies that reported data only on secondary outcomes (Hillmen et al., 2007; Hillmen et al., 2011; Foà et al., 2014; Burger et al., 2015; Österborg et al., 2016; Flinn et al., 2018; Huang et al., 2018; Moreno et al., 2019; Sharman et al., 2021) (Figure 1). Baseline characteristics of the studies with extractable outcomes are presented in Supplementary Table S1.

Relevant studies that were included in our primary analysis provided data on 11,660 patients (6,178 being assigned to the treatment arm and 5,482 to the control arm). The median age of participants ranged from 58 to 74 years and the mean percentage of males was 68.1%. Patients with relapsed or refractory CLL/SLL were included in 11 studies (Robak et al., 2010; Elter et al., 2011; Byrd et al., 2014; Furman et al., 2014; Chanan-Khan et al., 2016; Jones et al., 2017; Robak et al., 2017; Zelenetz et al., 2017; Seymour et al., 2018; Ghia et al., 2020; Byrd et al., 2021), while 11 studies included only primarily treatment naïve patients (Hallek et al., 2010; Geisler et al., 2014; Goede et al., 2014; Hillmen et al., 2015; Eichhorst et al., 2016; Woyach et al., 2018; Fischer et al., 2019; Shanafelt et al., 2019; Sharman et al., 2020; Wierda et al., 2021; Langerbeins et al., 2022). Two RCTs included both of these patient populations (Michallet et al., 2018; Burger et al., 2019). The median number of prior therapies ranged from 0 to 3. Only two studies reported a median of three prior therapies and both of them evaluated the PI3K inhibitor, idelalisib (Furman et al., 2014; Jones et al., 2017). Additionally, we retrieved one study of consolidation (Wendtner et al., 2004) and six studies on maintenance treatment (van Oers et al., 2015; Greil et al., 2016; Chanan-Khan et al., 2017; Fink et al., 2017; Dartigeas et al., 2018; Jindal et al., 2021).

In Table 1 we present the pooled estimates of the cumulative incidence of severe infections categorized by mechanism of action of the drug. We also present a list of the used drugs in Table 1. The Egger’s test for publication bias showed no evidence of small study effects (bias = -0.1, *p* = 0.872), while the heterogeneity among the studies ranged from moderate to considerable (I^2^: 31.8–97.9%). Also, before performing further analyses, we evaluated the baseline status of patients. The studies that we included in our analysis used patients that fulfilled the International Workshop on Chronic Lymphocytic Leukemia (iwCLL) criteria for CLL treatment (Hallek et al., 2018). Interestingly, the studies we retrieved allowed the inclusion of patients with a performance-status that ranged from 0 to 2 on the Eastern Cooperative Oncology Group (ECOG) scale (Oken et al., 1982). The ECOG performance-status scales from 0 to 5, and a status of 0 indicates the absence of symptoms while higher scores suggest increased disability. Of note, one study (Furman et al., 2014) did not report ECOG performance status and allowed the inclusion of patients with a score of less than six on the Cumulative Illness Rating Scale (CIRS) (Extermann et al., 1998). Therefore, we assume that the baseline status of the included patients is comparable.

**TABLE 1:** Pooled cumulative incidence of severe infections.

Drug Class	Pooled cumulative incidence (%)	95% CI
BTKi	19.86	16.03–23.98%
PI3Ki	30.89	24.33–37.85%
2nd generation anti-CD20	13.46	10.52–16.70%
Rituximab	19.85	16.06–23.94%
Anti-BCL2	17.49	13.92–21.36%
Lenalidomide	13.33	7.83–19.90%
anti-CD52	45.09	7.46–86.25%

BCL2, B-cell lymphoma-2, protein; BTKi, Bruton tyrosine kinase inhibitor; CI, confidence interval, PI3Ki, Phosphoinositide 3-kinase inhibitor.

Drug names list: Anti-BCL2: venetoclax, anti-CD52: alemtuzumab, anti-CD20: obinutuzumab, ofatumumab, rituximab BTKi: acalabrutinib, ibrutinib, PI3Ki: idelalisib.

### 3.1 BTKi-based regimens

**FIGURE 2:**
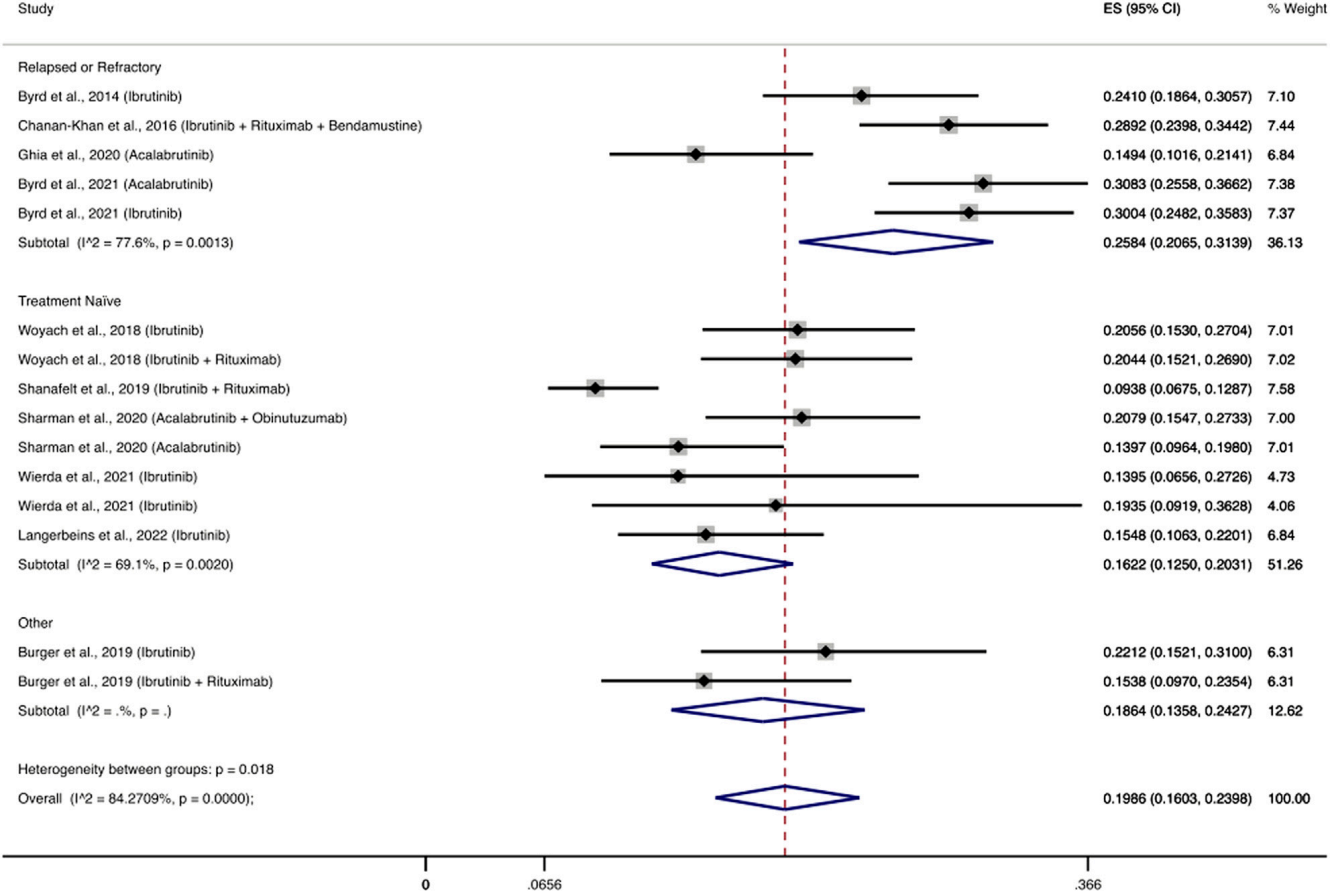
Cumulative incidence rate of severe infections BTK inhibitors. Individual and combined estimates of the incidence of severe infections for patients treated with BTK inhibitors with 95% confidence intervals. ES: Effect Size (Cumulative incidence).

Ten RCTs evaluated the use of BTK inhibitors among 2,672 patients and reported 558 patients with severe infections (Byrd et al., 2014; Chanan-Khan et al., 2016; Woyach et al., 2018; Burger et al., 2019; Shanafelt et al., 2019; Ghia et al., 2020; Sharman et al., 2020; Byrd et al., 2021; Wierda et al., 2021; Langerbeins et al., 2022). The pooled cumulative incidence of severe infections was 19.86% (95% CI: 16.03–23.98%) (Figure 2). The most common infections were pneumonia (2.2–10.5%), upper respiratory tract infections (0.4–2.9%), and urinary tract infections (0–4%). Notably, eight RCTs evaluated a BTKi as a monotherapy (Byrd et al., 2014; Woyach et al., 2018; Burger et al., 2019; Ghia et al., 2020; Sharman et al., 2020; Byrd et al., 2021; Wierda et al., 2021; Langerbeins et al., 2022), among 1,570 patients and reported 352 patients that developed severe infections. The pooled cumulative incidence was 20.7% (95% CI: 16.48–25.26%) (Supplementary Figure S1). Additionally, five RCTs evaluated a BTKi as part of a combination therapy with obinutuzumab or rituximab or bendamustine-rituximab, and reported 206 patients with severe infections among 1,102 patients (Chanan-Khan et al., 2016; Woyach et al., 2018; Burger et al., 2019; Shanafelt et al., 2019; Sharman et al., 2020). Consequently, the cumulative incidence of infections in this patient population was 18.54% (95% CI: 11.37–26.96%) (Supplementary Figure S2)).

**FIGURE 3:**
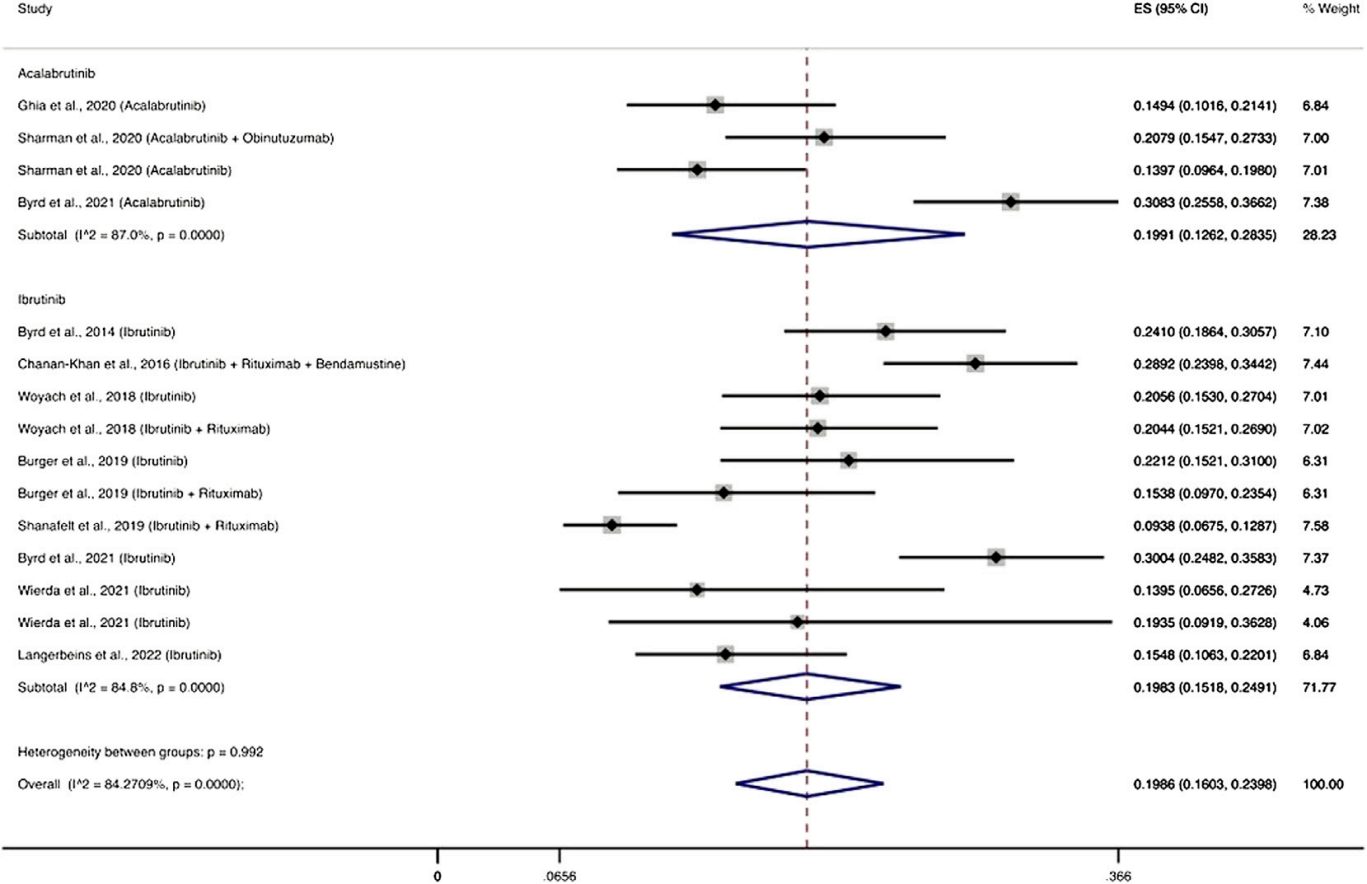
BTKi compounds and the cumulative incidence of severe infections. Individual and combined estimates of the incidence of severe infections for patients treated with acalabrutinib and ibrutinib with 95% confidence intervals. ES: Effect Size (Cumulative incidence).

Three RCTs (Ghia et al., 2020; Sharman et al., 2020; Byrd et al., 2021) evaluated the use of acalabrutinib among 777 patients and reported 167 patients with severe infections. The pooled incidence of severe infections was 19.91% (95% CI, 12.62–28.35%). Eight RCTs (Byrd et al., 2014; Chanan-Khan et al., 2016; Woyach et al., 2018; Burger et al., 2019; Shanafelt et al., 2019; Byrd et al., 2021; Wierda et al., 2021; Langerbeins et al., 2022) evaluated the use of ibrutinib among 1,895 patients and reported 391 patients with severe infections, with a pooled incidence rate of severe infections of 19.83% (95% CI, 15.18–24.91%) (Figure 3).

### 3.2 PI3K δ inhibitor and anti-CD52 regimens

#### 3.2.1 PI3K-δ inhibitors

**FIGURE 4:**
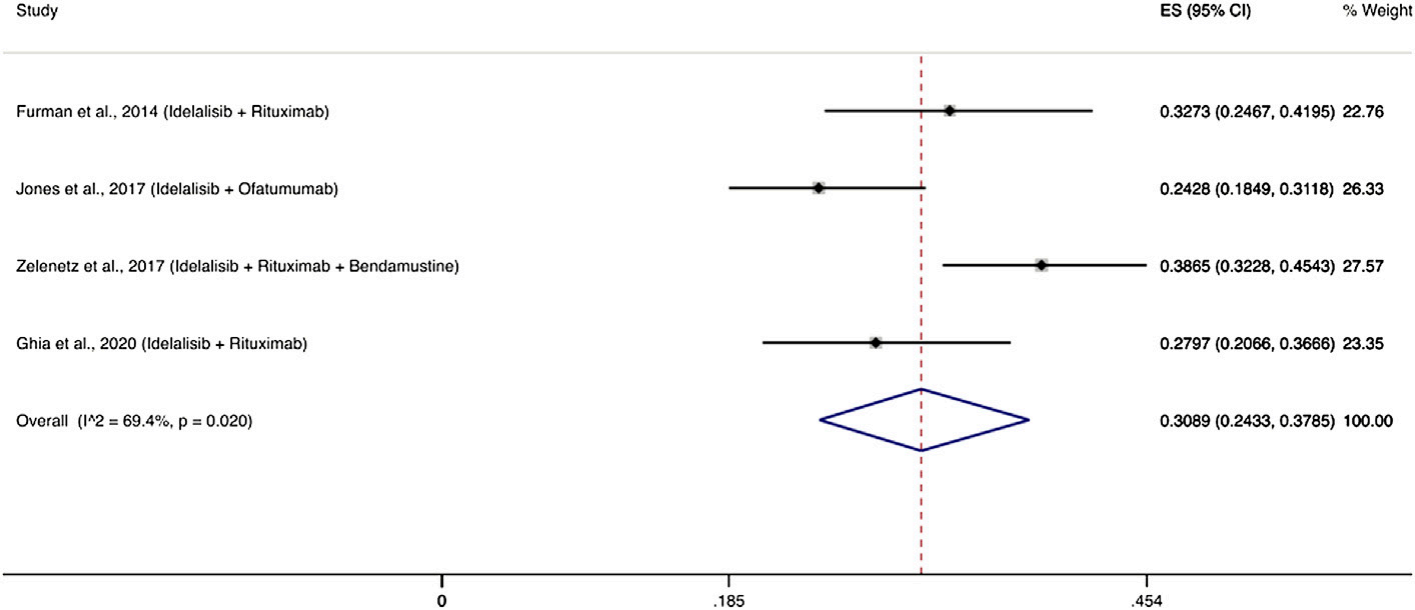
Cumulative incidence rate of severe infections for PI3K δ inhibitors. Individual and combined estimates of the incidence of severe infections for patients treated with PI3K inhibitors with 95% confidence intervals. ES: Effect Size (Cumulative incidence).

Four RCTs evaluated PI3K inhibitors and specifically idelalisib among 608 patients with relapsed/refractory disease and reported 191 patients with severe infections (Furman et al., 2014; Jones et al., 2017; Zelenetz et al., 2017; Ghia et al., 2020). The cumulative incidence of infections was 30.89% (95% CI: 24.33–37.85%) (Figure 4). The most frequent infections in these studies were pneumonia (6–13%), febrile neutropenia (5–24%) and sepsis (1.6–5.7%). Two studies reported *P. jirovecii* pneumonia in 3 and 5% of patients who received PI3K inhibitors (Furman et al., 2014; Jones et al., 2017).

#### 3.2.2 Anti-CD52 monoclonal antibody-based therapies

Only three RCTs evaluated alemtuzumab, a monoclonal antibody that targets CD-52 among only 308 patients and reported 118 patients with severe infections (Wendtner et al., 2004; Elter et al., 2011; Geisler et al., 2014). The pooled cumulative incidence of infections was 45.09% (95% CI: 7.46–86.25%). The license of alemtuzumab for CLL/SLL was withdrawn in 2012 (Hallek and Al-Sawaf, 2021).

### 3.3 Anti-CD20 monoclonal antibody-based therapies

#### 3.3.1 First generation anti-CD20 monoclonal antibody-based therapies

Fourteen RCTs evaluated the established anti-CD20 monoclonal antibody rituximab (Hallek et al., 2010; Robak et al., 2010; Furman et al., 2014; Goede et al., 2014; Chanan-Khan et al., 2016; Eichhorst et al., 2016; Greil et al., 2016; Zelenetz et al., 2017; Dartigeas et al., 2018; Michallet et al., 2018; Seymour et al., 2018; Woyach et al., 2018; Shanafelt et al., 2019; Ghia et al., 2020) among 3,395 patients and reported 729 patients with severe infections. The cumulative incidence of severe infections was 19.85% (95% CI: 16.06–23.94%) (Supplementary Figure S3).

#### 3.3.2 Second generation anti-CD20 monoclonal antibodies

**FIGURE 5:**
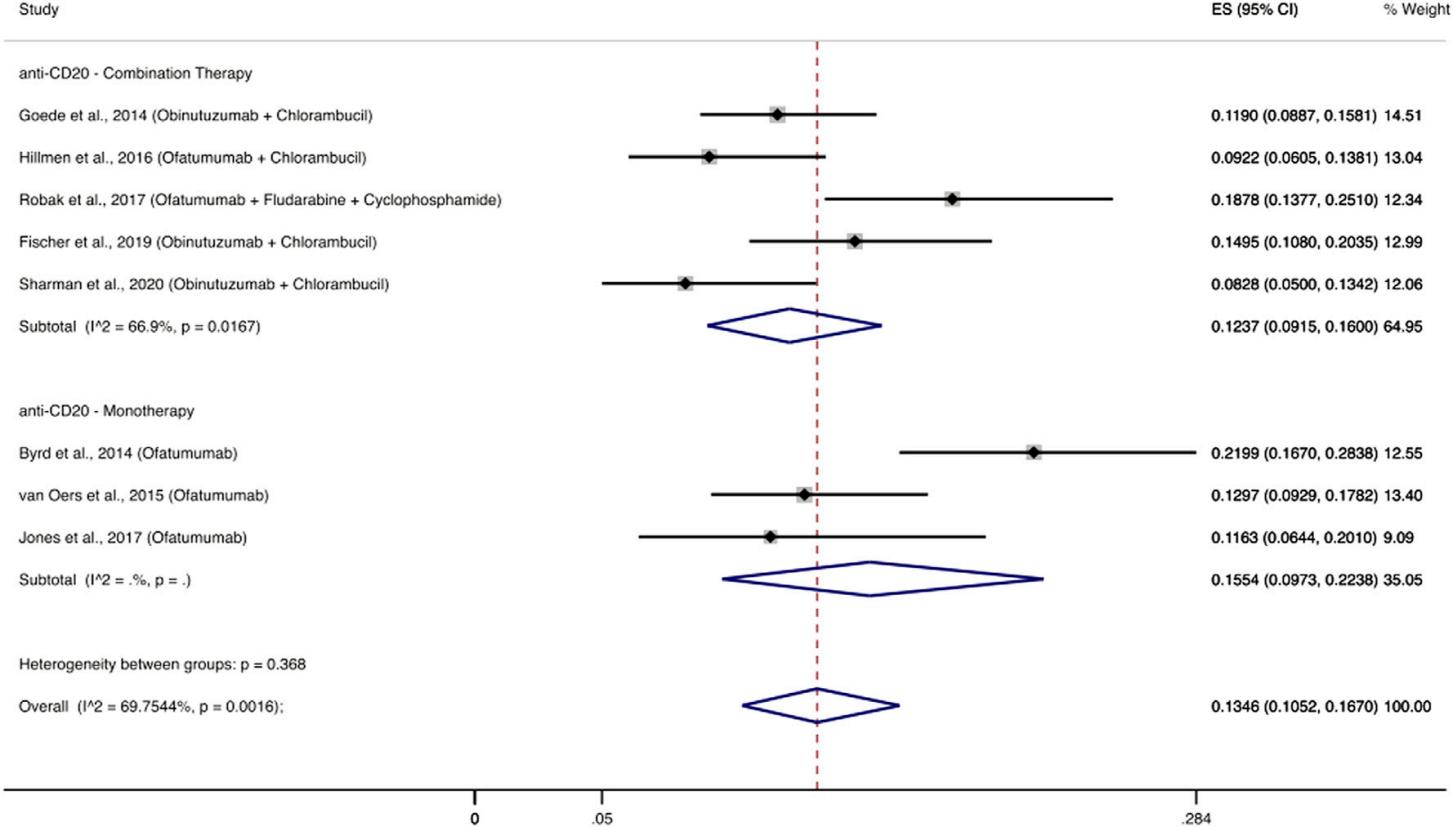
Cumulative incidence rate of severe infections for second generation anti-CD20 monoclonal antibodies. Individual and combined estimates of the incidence of severe infections for patients treated with second generation anti-CD20 monoclonal antibodies with 95% confidence intervals. ES: Effect Size (Cumulative incidence).

Eight RCTs evaluated the use of second generation anti-CD20 monoclonal antibodies (ofatumumab, obinutuzumab) among 1,633 patients and reported 223 patients with severe infections (Byrd et al., 2014; Goede et al., 2014; Hillmen et al., 2015; van Oers et al., 2015; Jones et al., 2017; Robak et al., 2017; Fischer et al., 2019; Sharman et al., 2020). The cumulative incidence of severe infections among these patients was 13.46% (95% CI: 10.52–16.70%). Infectious complications that appeared most frequently were pneumonia (1.8–16%), febrile neutropenia (2–5.9%), and sepsis (0.9–3%). Three RCTs (Byrd et al., 2014; van Oers et al., 2015; Jones et al., 2017) evaluated a second generation anti-CD20 monoclonal antibody as monotherapy and the cumulative incidence of severe infections was 15.54% (95% CI: 9.73–22.38%), while five RCTs (Goede et al., 2014; Hillmen et al., 2015; Robak et al., 2017; Fischer et al., 2019; Sharman et al., 2020) used combination treatments with a cumulative incidence of severe infections at 12.37% (95% CI: 9.15–16%) (Figure 5).

### 3.4 BCL-2 inhibitor

Two RCTs evaluated the BCL-2 inhibitor, venetoclax, among 406 patients, and reported 76 patients that developed severe infections (Seymour et al., 2018; Fischer et al., 2019). Accordingly, the cumulative incidence of severe infections was 17.49% (95% CI: 13.92–21.36%) (Supplementary Figure S4). Of note, both RCTs that evaluated venetoclax-based treatments utilized regimens that also included anti-CD20 monoclonal antibodies, as venetoclax was not administered as a monotherapy.

### 3.5 Lenalidomide

Three RCTs included the immunomodulator lenalidomide as a maintenance therapy (Chanan-Khan et al., 2017; Fink et al., 2017; Jindal et al., 2021). Lenalidomide has not been approved for treatment in CLL and is only used in clinical trials. From these trials, we retrieved 35 out of 238 patients with severe infections. The pooled cumulative incidence was 13.33% (95% CI: 7.83–19.9%) (Supplementary Figure S5).

### 3.6 Treatment naïve vs. relapsed/refractory disease

We performed a subgroup analysis between treatment naïve patients and patients with relapsed or refractory disease. In the analysis of patients that received BTKi-based treatment we included nine studies with 13 regimens (Byrd et al., 2014; Chanan-Khan et al., 2016; Woyach et al., 2018; Shanafelt et al., 2019; Ghia et al., 2020; Sharman et al., 2020; Byrd et al., 2021; Wierda et al., 2021; Langerbeins et al., 2022) and excluded a study that had a mixed patient population (Burger et al., 2019). Patients with relapsed or refractory disease had a pooled cumulative incidence of severe infections of 25.84% (95% CI: 20.65–31.39%), while the respective cumulative incidence for treatment naïve patients was 16.22% (95% CI: 12.5–20.31%) (Figure 2). Regarding the rest of the drug classes, sub-grouping by treatment setting did not show any significant results (data not shown) or the number of studies was limited (Supplementary Table S2).

### 3.7 Secondary outcomes

For the secondary outcomes we evaluated the incidence of severe pneumonia, febrile neutropenia and sepsis. We assessed these incidences across the same agents that we studied for the primary outcome. Secondary analysis results are presented in Table 2. For BTKi-based treatments, the estimated cumulative incidence of pneumonia was 6.33% (95% CI: 4.92–7.90%). For the same drug class the cumulative incidence of febrile neutropenia and sepsis was 2.68% (95% CI: 1.51–4.12%) and 1.43% (95% CI: 0.73–2.33%), respectively.

**TABLE 2:** Secondary outcomes.

Drug Class	Pneumonia	Febrile neutropenia	Sepsis
BTKi	6.33%	2.68%	1.43%
	(4.92–7.90%)	(1.51–4.12%)	(0.73–2.33%)
PI3Ki	8.45%	10.80%	4.48%
	(4.88–12.83%)	(4.17–19.92%)	(2.17–7.51%)
2nd generation anti-CD20	4.92%	2.86%	0.9%
	(2.93–7.35%)	(2.01–3.85%)	(0.4–1.53%)
Rituximab	6%	5.83	1.86%
	(4.37–7.86%)	(3.65–8.45%)	(1.13–2.73%)
anti-BCL2	4.67%	4.40%	1.53%
	(2.78–6.98%)	(2.57–6.66%)	(0.49–3.03%)
Lenalidomide	4.97%	1.91%	2.55%
	(2.31–8.45%)	(0.65–5.47%)	(1–6.37%)
anti-CD52	3.01%	4.16	2.72%*
	(0.5–67.4%)	(2.15–6.74%)	(1.06–6.79%)

## 4 Discussion

Treatment of CLL/SLL is transitioning to an approach that focuses on the use of targeted therapies (Burger and O’Brien, 2018; Burger, 2020; Hallek and Al-Sawaf, 2021). In our systematic review and meta-analysis, we estimated the cumulative incidence of severe infections in patients with CLL/SLL treated with targeted treatments. Depending on the treatment agent, the pooled estimate of the cumulative incidence for severe infections ranged from 13.33 to 45.09%. Patients who received lenalidomide had the lowest and those who received alemtuzumab had the highest. Notably, patients who were treated with BTKi regimens had higher incidence of severe infections in the relapsed or refractory treatment setting, while those who received PI3K inhibitors developed severe infections at a substantial rate of 30.89% and had the highest incidence of pneumonia, febrile neutropenia and sepsis across the agents studied. Additionally, we found a trend of lower incidence for infections, pneumonia, febrile neutropenia and sepsis among patients who were treated with second generation anti-CD20 monoclonal antibodies compared with rituximab.

Patients who received PI3K inhibitors had a significant incidence of severe infections at 30.89% and the use of PI3K inhibitors was associated with the highest incidence of severe pneumonia, febrile neutropenia, and sepsis (8.45, 10.80 and 4.48%, respectively).

Generally, PI3K inhibitors are reserved for patients who have already been treated with a BTKi and venetoclax. They are considered an alternative because of their lower efficacy in comparison with BTK inhibitors and because of their infectious/autoimmune complications, such as *Pneumocystis* pneumonia and cytomegalovirus infection (Hoellenriegel et al., 2011; Reinwald et al., 2018; Burger, 2020). In real-world, the use of *Pneumocystis jirovecii* prophylaxis is recommended by the manufacturer for patients treated with idelalisib during treatment-period and for 2 to 6 months after treatment cessation if infection risk persists (Zydelig, 2018). In the included studies, despite the use of prophylaxis for *Pneumocystis jirovecii* and cytomegalovirus, we found that severe infections, pneumonia, febrile neutropenia and sepsis still appeared at a remarkable rate. Interestingly, patients who received PI3K inhibitors only had relapsed/refractory disease and the highest median number of prior therapies compared with other agents of our meta-analysis (Furman et al., 2014; Jones et al., 2017; Zelenetz et al., 2017; Ghia et al., 2020). Thus, advanced disease setting and the use of two or more prior therapies most likely contributed to the observed incidences of these infectious manifestations. Future studies need to investigate additional prevention strategies for patients who receive PI3K inhibitors and assess the infectious complications of these agents in the treatment naïve setting.

Almost one out five patients (19.86%) who received BTKi-based treatments developed severe infections, confirming the risk that was shown in previous studies (Tillman et al., 2018; Holowka et al., 2021). Markedly, pneumonia presented with the second highest cumulative incidence (6.33%) behind PI3K inhibitors (10.80%) across the agents we studied. Interestingly, BTK inhibitors affect the immune system by hampering innate immunity and by disrupting the B-cell receptor (BCR) signaling pathway, leading to impaired development and function of B-lymphocytes (Weber et al., 2017; Maffei et al., 2020; Holowka et al., 2021; Palma et al., 2021). In this regard, it is reasonable to assume that one aspect that contributes to the development of severe infections and pneumonia is the mechanism of action of BTK inhibitors.

The incidence of severe infections among patients treated with BTK inhibitors was remarkably higher among relapsed or refractory patients (25.84%) compared with treatment naïve patients (16.2%). CLL/SLL disease progression and the use of prior lines of treatment most likely have considerable impact on the development of severe infections in patients who receive BTKi-based regimens. Therefore, the immunodeficiency caused by relapsed/refractory disease along with the mechanism of action of BTKis presumably promote development of severe infections. Future studies are needed so that we can define precise patient characteristics that predispose to infectious complications patients with relapsed/refractory disease that receive BTKi treatments. Subsequently, specific antimicrobial prevention strategies should be investigated for patients with relapsed/refractory CLL/SLL.

Regarding anti-CD20 agents, we found an incidence of severe infections ranging from 13.46 to 19.85% which is similar to the incidence reported in anti-neutrophil cytoplasmic antibody-associated vasculitides (15.4%) (Thery-Casari et al., 2020). Intriguingly, the newer anti-CD20 agents numerically displayed lower incidence rates of severe infections, pneumonia, febrile neutropenia, and sepsis compared to rituximab (Table 2). Overall, treatment regimens based on second generation anti-CD20 monoclonal antibodies had the lowest pooled cumulative incidence of sepsis (0.9%) among every agent we evaluated (Table 2). These data provide further supportive evidence that for the treatment of CLL/SLL or even other diseases, second generation anti-CD20 monoclonal antibodies could eventually replace rituximab, as they have similar or even better efficacy and safety profiles (Burger and O’Brien, 2018).

This systematic review and meta-analysis had some limitations. Many of the studies we retrieved, reported only some of the outcomes of interest and the majority of trials did not report the timing of infectious events, highlighting the need for a universal way of reporting safety results for RCTs. Data regarding the rate of severe infections in patients treated with BCL-2 inhibitors, alemtuzumab and lenalidomide were limited, since few RCTs were included (2,3 and 3 respectively). Additionally, the nature of treatment in CLL/SLL and other hematological malignancies most frequently requires the use of combination treatments, so assessing relative impact of treatment is challenging. Regarding anti-CD20 monoclonal antibody-based regimens, we observed a diverse landscape of chemotherapy combinations which included both intensive (fludarabine, cyclophosphamide) and mild (chlorambucil) treatment options. Such diversity in treatment regimens could have affected the results shown.

In conclusion, this meta-analysis confirms the high rate of severe infections across different targeted regimens utilized for the treatment of CLL/SLL (13–45%). Infections in patients with relapsed/refractory disease tend to appear at a higher incidence compared with patients that are primarily treatment naïve. On this regard, we should be even more vigilant when treating patients who have already received a prior line of treatment. Additionally, this analysis outlines differences in infectious complications between different regimens for patients with CLL/SLL. These differences should be taken into account when selecting a targeted agent. Future clinical trials that utilize and compare these targeted agents are needed to further understand patient risk factors that increase the risk of severe infections and evaluate infection prevention and antimicrobial stewardship protocols.

## Data Availability

The original contributions presented in the study are included in the article/Supplementary Materials, further inquiries can be directed to the corresponding author.

## Abbreviations

BCL-2, B-cell lymphoma-2 protein, BTK: bruton tyrosine kinase, BTKi: bruton tyrosine kinase inhibitor, CLL: chronic lymphocytic leukemia, PI3K: phosphoinositide 3-kinase, RCT: randomized controlled trial, SLL: small lymphocytic lymphoma.
