# Supplementary material for: Targeted therapies in CLL/SLL and the cumulative incidence of infection: A systematic review and meta-analysis
Source: Front Pharmacol. 2022 Sep 14;13:989830. doi: 10.3389/fphar.2022.989830 (PMC9515578; doi:10.3389/fphar.2022.989830)
Supplement: Supplementary file 1 [file DataSheet1.pdf]

Supplementary Appendix

Supplement to: Targeted therapies in CLL/SLL and the cumulative incidence of infections: a systematic review and meta-analysis

Contents

Table S1: Baseline characteristics

Table S2: Treatment naive versus Relapsed/Refractory

Figure S1: Cumulative incidence rate of severe infections with BTKi monotherapy

Figure S2: Cumulative incidence rate of severe infections for BTKi not-monotherapy

Figure S3: Cumulative incidence rate of severe infections for Rituximab

Figure S4: Cumulative incidence rate of severe infections for BCL2 inhibitors

Figure S5: Cumulative incidence rate of severe infections for lenalidomide

References

Table S1 Baseline characteristics

Study	Study Name	Phase	Follow-up duration (months)	Treatment setting	No of patients	No of control	Treatment regimen	Control regimen	Median age	Median age control	Rob 2 Bias
**Ghia, et al 2020** (1)	ASCEND	3	16.1	R/R	154	153	ACA	PC	68	67	Low
**Sharman, et al 2020** (2)	ELEVATE-TN	3	28.3	TN	178	179	ACA + O	O + Chl	70	71	Low
**Sharman, et al 2020** (2)	ELEVATE-TN	3	28.3	TN	179	179	ACA	O + Chl	70	71	Low
**Byrd, et al 2021** (3)	N/A	3	40.9	R/R	266	263	ACA	Ibr	66	65	Low
**Elter, et al 2011** (4)	N/A	3	29.5	R/R	164	165	Ale + F	F	N/A	N/A	Low
**Geisler, et al 2014** (5)	HOVON68	3	42.8	TN	133	136	Ale + F + C	F + C	N/A	N/A	Low
**Wendtner, et al 2004** (6)	GCLLSG	3	21.4	Consolidation 1L	11	10	Ale	Observation	60	60	Low
**Michallet, et al 2018** (7)	MABLE	3	23.5	TN, R/R	177	178	R + B	R + Chl	73	72	Low
**Burger, et al 2019** (8)	N/A	2	36	TN, R/R	104	104	Ibr	Ibr + R	65	65	Low
**Shanafelt, et al 2019** (9)	E1912	3	33.6	TN	352	158	Ibr + R	F + C + R	N/A	N/A	Low
**Woyach, et al 2018** (10)	Alliance A041202	3	38	TN	180	176	Ibr	R + B	71	70	Low
**Woyach, et al 2018** (10)	Alliance A041203	3	38	TN	181	176	Ibr + R	R + B	71	70	Low
**Langerbeins, et al 2022** (11)	CLL12	3	31	TN	155	158	Ibr	Placebo	64	64	Low
**Chanan-Khan, et al 2016** (12)	HELIOS	3	17	R/R	287	287	Ibr + R + B	Placebo + R + B	64	63	Low
**Byrd, et al 2014** (13)	RESONATE	3	9.4	R/R	195	191	Ibr	OFA	67	67	Low
**Wierda, et al 2021** (14)	CAPTIVATE	2	31.3	TN	43	43	Ibr	Placebo	58	58	Low
**Wierda, et al 2021** (14)	CAPTIVATE	2	31.3	TN	31	32	Ibr	Ibr + VEN	58	58	Low
**Furman, et al 2014** (15)	N/A	3	18	R/R	110	107	IDELA + R	Placebo + R	71	71	Low

Study	Study Name	Phase	Follow-up duration (months)	Treatment setting	No of patients	No of control	Treatment regimen	Control regimen	Median age	Median age control	Rob 2 Bias
**Jones, et al 2017** (16)	N/A	3	16.1	R/R	173	86	IDELA + OFA	OFA	68	67	Low
**Zelenetz, et al 2017** (17)	N/A	3	14	R/R	207	209	IDELA + R + B	Placebo + R + B	62	64	Low
**Fink, et al 2017** (18)	CLLM1	3	17.9	M 1L	56	29	L	Placebo	64	64	Low
**Chanan-Khan, et al 2017** (19)	CONTINUUM	3	31.5	M 2L	157	154	L	Placebo	63	63	Low
**Jindal, et al 2021** (20)	N/A	2	22	M 1L	25	15	L	Observation	60	62	Low
**Goede, et al 2014** (21)	CLL11	3		TN	336	321	O + Chl	R + Chl	74	73	Low
**Hillmen, et al 2016** (22)	COMPLEMENT1	3	28.9	TN	217	227	OFA + Chl	Chl	69	70	Low
**Robak, et al 2017** (23)	COMPLEMENT2	3	34	R/R	181	178	OFA + F + C	F + C	62	61	Low
**van Oers, et al 2015** (24)	PROLONG	3	19.1	M 2L	239	241	OFA	Observation	64	64.5	Low
**Hallek, et al 2010** (25)	CLL8	3		TN	396	404	F + C + R	F + C	61	61	Low
**Robak, et al 2010** (26)	REACH	3	25	R/R	274	272	F + C + R	F + C	63	62	Low
**Greil, et al 2016** (27)	AGMT CLL-8a Mabtenance	3	33.7	M	134	129	R	Observation	63	63	Low
**Eichhorst, et al 2016** (28)	CLL10	3	37.1	TN	279	278	F + C + R	R + B	62	61	Low
**Dartigeas, et al 2018** (29)	CLL 2007 SA	3	47.7	M 1L	198	207	R	Observation	mean 71.7	71.1	Low
**Fischer, et al 2019** (30)	CLL14	3	28.1	TN	212	214	VEN + O	Chl + O	72	71	Low
**Seymour, et al 2018** (31)	MURANO	3	23.8	R/R	194	188	VEN + R	R + B	64.5	66	Low
**Hillmen, et al 2007** (32)	CAM 307	3	24.6	TN	147	147	Ale	Chl	59	60	Low
**Huang, et al 2018** (33)	N/A	3	17.8	R/R	104	52	Ibr	R	65	67	Low
**Burger, et al 2015** (34)	RESONATE-2	3	18.4	TN	135	132	Ibr	Chl	73	72	Low

Study	Study Name	Phase	Follow-up duration (months)	Treatment setting	No of patients	No of control	Treatment regimen	Control regimen	Median age	Median age control	Rob 2 Bias
**Moreno, et al 2019** (35)	iLLUMINATE	3	31.3	TN	113	115	Ibr + O	Chl + O	70	72	Low
**Foa, et al 2014** (36)	N/A	2	34.2	M 1L	34	32	R	Observation	70	70	Low
**Hillmen, et al 2011** (37)	N/A	2	29	R/R	26	26	R + F + C + Mi	F + C + Mi	66	68	Low
**Sharman, et al 2021** (38)	GENUINE	3	41.6	R/R	59	58	Ibr + Ublituximab	Ibr	66	67	Low
**Osterborg, et al 2016** (39)	N/A	3	12	R/R	79	43	OFA	PC	62	63	Low
**Flinn, et al 2018** (40)	DUO	3	22.4	R/R	158	155	Duvelisib	OFA	69	69	Low

Abbreviations: 1L, first line, 2L, second line, ACA, acalabrutinib, Ale, alemtuzumab, B, bendamustine, C, cyclophosphamide, Chl, chlorambucil, F, fludarabine, Ibr, ibrutinib, IDELA, idelalisib, L, lenalidomide, M, maintenance, Mi, mitoxantrone, O, Obinutuzumab, OFA, ofatumumab, PC, physician's choice, R, rituximab, R/R, relapsed or refractory, TN, treatment naïve, VEN, venetoclax

Table S2 Treatment naive versus Relapsed/Refractory

Drug class	Treatment naïve	Relapsed/Refractory
**BTKi**	16.22%<br>(12.50 - 20.31%)	25.84%<br>(20.65 - 31.39%)
**2nd generation anti-CD20**	11.11%<br>(8.54 - 13.97%)	17.97%<br>(12.91 - 23.65%)
**Rituximab**	21.02%<br>(13.20 - 30.09%)	21.28%<br>(18.88 - 23.78%)
**PI3Ki**	N/A	30.89%<br>(24.33 - 37.85%)

Figure S1 Cumulative incidence rate of severe infections with BTKi monotherapy

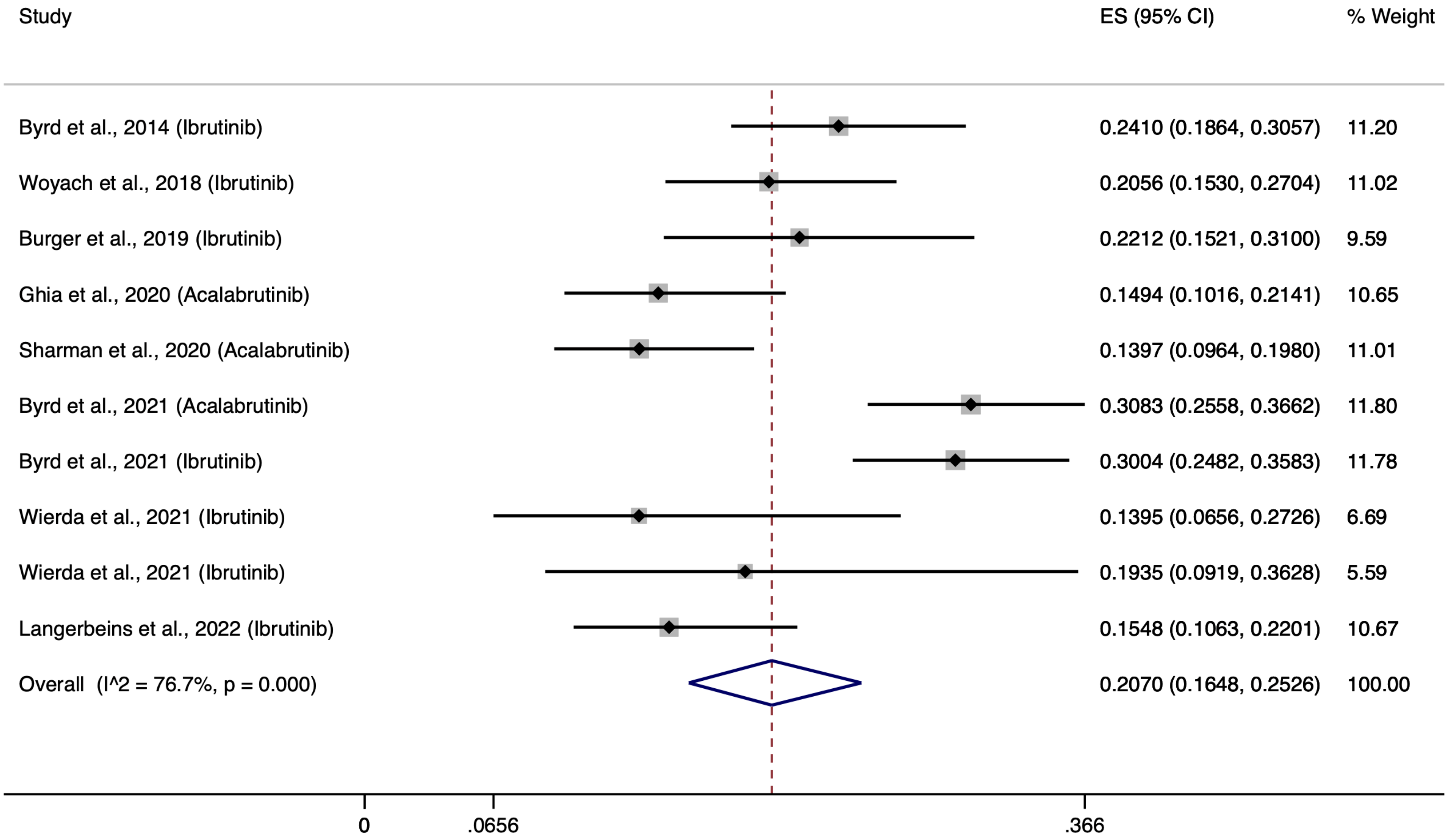

Figure S2 Cumulative incidence rate of severe infections for BTKi not-monotherapy

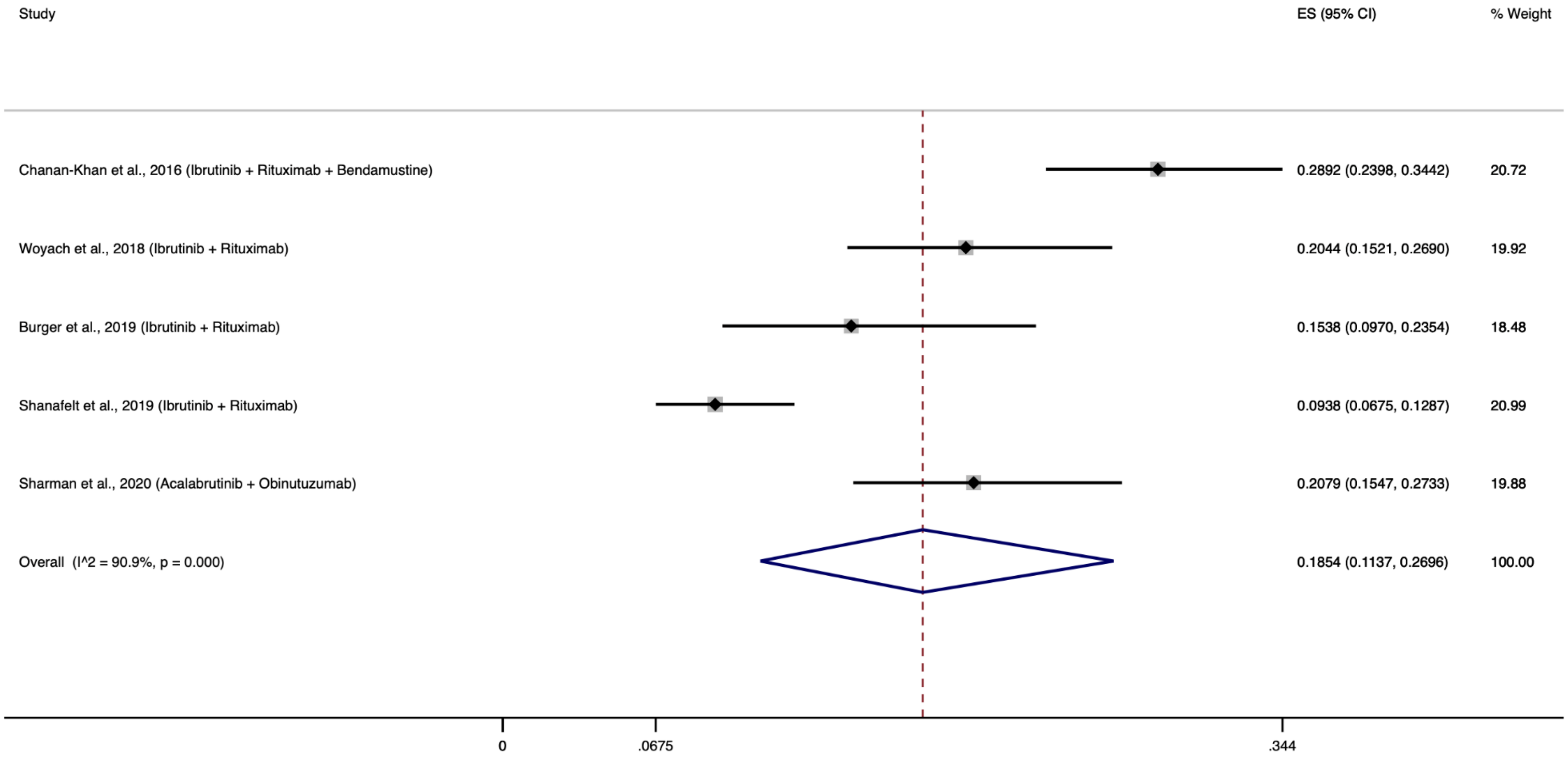

Figure S3 Cumulative incidence rate of severe infections for Rituximab

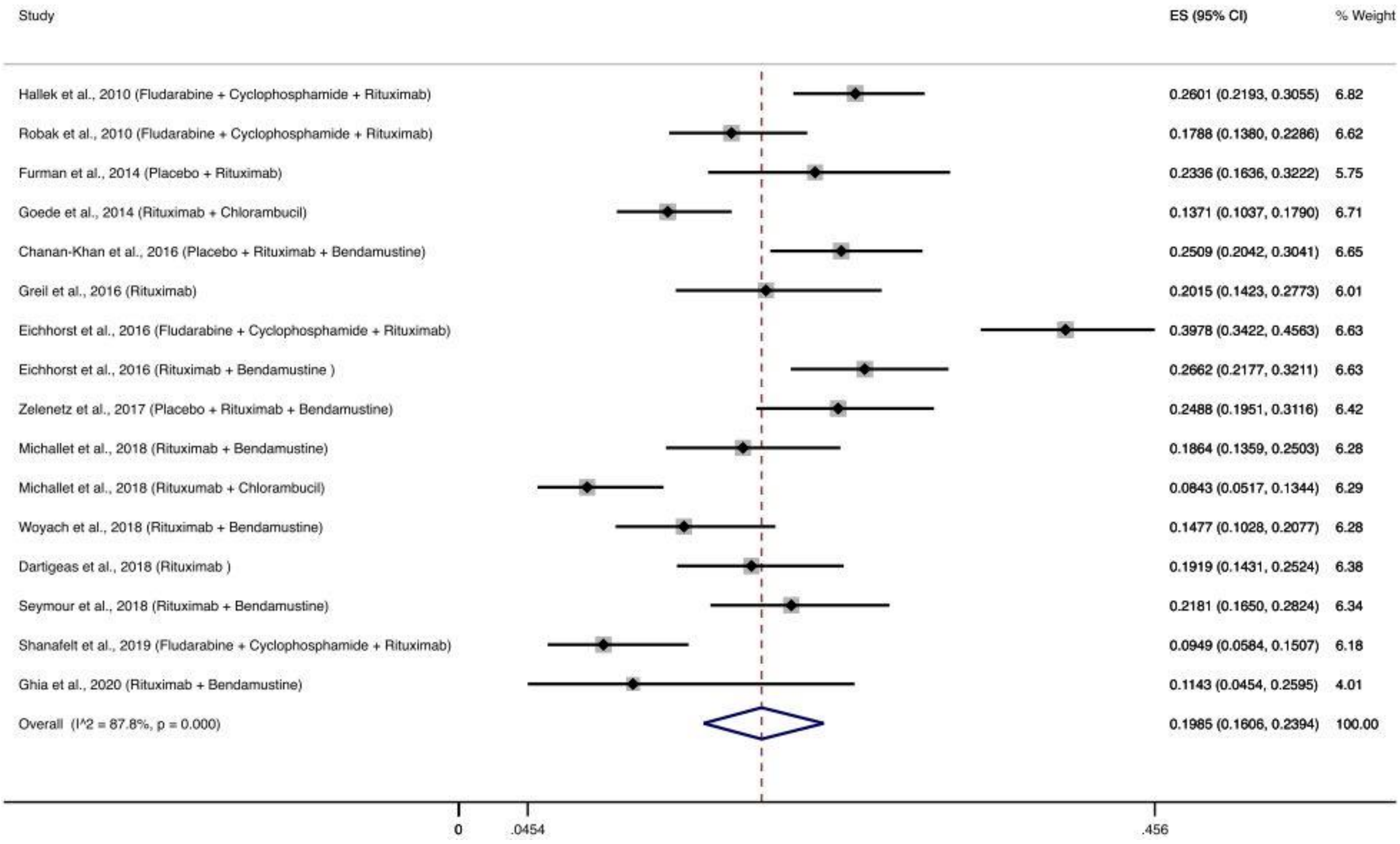

Figure S4 Cumulative incidence rate of severe infections for BCL2 inhibitors

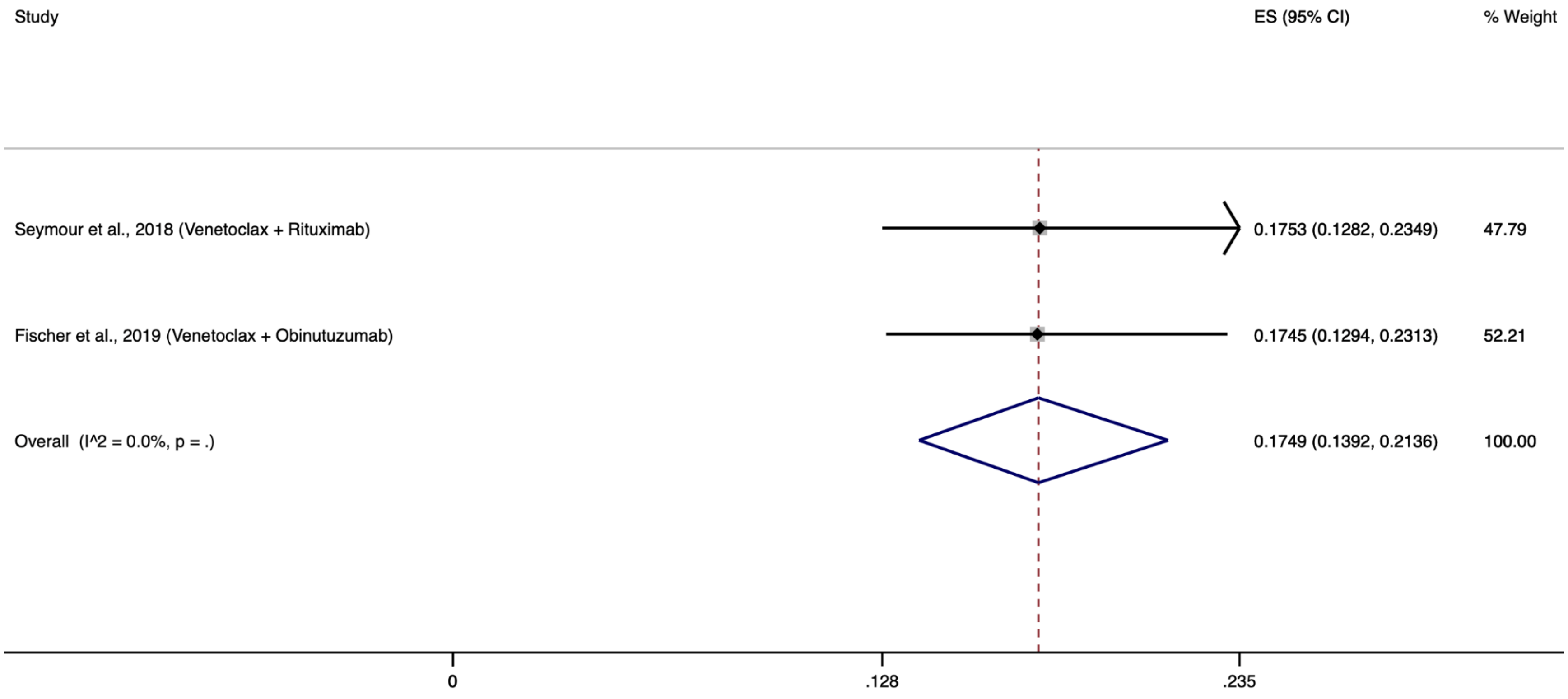

Figure S5 Cumulative incidence rate of severe infections for lenalidomide

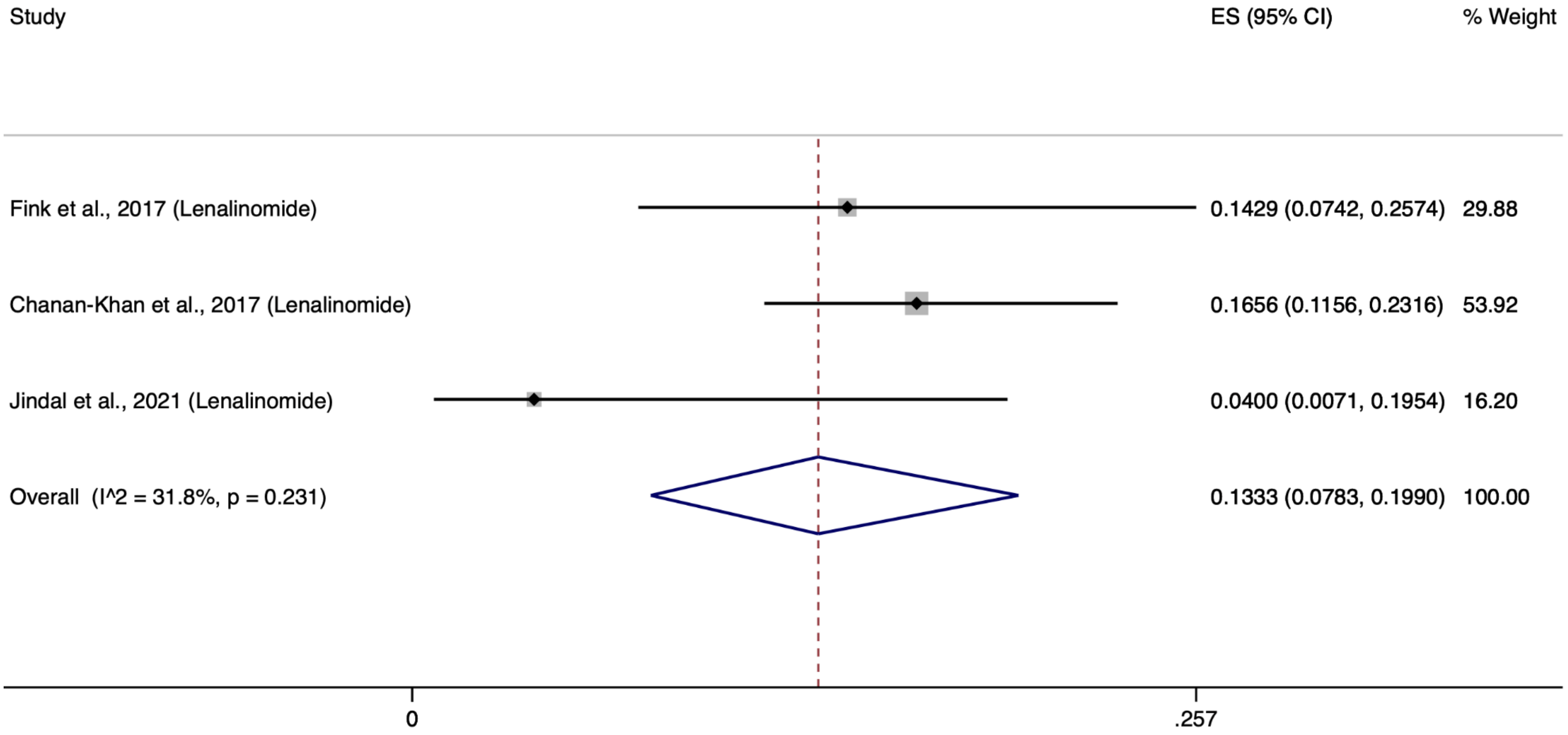

References

1. Ghia P, Pluta A, Wach M, Lysak D, Kozak T, Simkovic M, et al. ASCEND: Phase III, Randomized Trial of Acalabrutinib Versus Idelalisib Plus Rituximab or Bendamustine Plus Rituximab in Relapsed or Refractory Chronic Lymphocytic Leukemia. J Clin Oncol. 2020;38(25):2849-61.
2. Sharman JP, Egyed M, Jurczak W, Skarbnik A, Pagel JM, Flinn IW, et al. Acalabrutinib with or without obinutuzumab versus chlorambucil and obinutuzmab for treatment-naive chronic lymphocytic leukaemia (ELEVATE TN): a randomised, controlled, phase 3 trial. Lancet. 2020;395(10232):1278-91.
3. Byrd JC, Hillmen P, Ghia P, Kater AP, Chanan-Khan A, Furman RR, et al. Acalabrutinib Versus Ibrutinib in Previously Treated Chronic Lymphocytic Leukemia: Results of the First Randomized Phase III Trial. J Clin Oncol. 2021;39(31):3441-52.
4. Elter T, Gercheva-Kyuchukova L, Pylylpenko H, Robak T, Jaksic B, Rekhtman G, et al. Fludarabine plus alemtuzumab versus fludarabine alone in patients with previously treated chronic lymphocytic leukaemia: a randomised phase 3 trial. Lancet Oncol. 2011;12(13):1204-13.
5. Geisler CH, van T' Veer MB, Jurlander J, Walewski J, Tjønnfjord G, Itälä Remes M, et al. Frontline low-dose alemtuzumab with fludarabine and cyclophosphamide prolongs progression-free survival in high-risk CLL. Blood. 2014;123(21):3255-62.
6. Wendtner CM, Ritgen M, Schweighofer CD, Fingerle-Rowson G, Campe H, Jäger G, et al. Consolidation with alemtuzumab in patients with chronic lymphocytic leukemia (CLL) in first remission--experience on safety and efficacy within a randomized multicenter phase III trial of the German CLL Study Group (GCLLSG). Leukemia. 2004;18(6):1093-101.
7. Michallet AS, Aktan M, Hiddemann W, Ilhan O, Johansson P, Laribi K, et al. Rituximab plus bendamustine or chlorambucil for chronic lymphocytic leukemia: primary analysis of the randomized, open-label MABLE study. Haematologica. 2018;103(4):698-706.
8. Burger JA, Sivina M, Jain N, Kim E, Kadia T, Estrov Z, et al. Randomized trial of ibrutinib vs ibrutinib plus rituximab in patients with chronic lymphocytic leukemia. Blood. 2019;133(10):1011-9.
9. Shanafelt TD, Wang XV, Kay NE, Hanson CA, O'Brien S, Barrientos J, et al. Ibrutinib-Rituximab or Chemoimmunotherapy for Chronic Lymphocytic Leukemia. N Engl J Med. 2019;381(5):432-43.
10. Woyach JA, Ruppert AS, Heerema NA, Zhao W, Booth AM, Ding W, et al. Ibrutinib Regimens versus Chemoimmunotherapy in Older Patients with Untreated CLL. N Engl J Med. 2018;379(26):2517-28.
11. Langerbeins P, Zhang C, Robrecht S, Cramer P, Fürstenau M, Al-Sawaf O, et al. The CLL12 trial: ibrutinib vs placebo in treatment-naïve, early-stage chronic lymphocytic leukemia. Blood. 2022;139(2):177-87.
12. Chanan-Khan A, Cramer P, Demirkan F, Fraser G, Silva RS, Grosicki S, et al. Ibrutinib combined with bendamustine and rituximab compared with placebo, bendamustine, and rituximab for previously treated chronic lymphocytic leukaemia or small lymphocytic lymphoma (HELIOS): a randomised, double-blind, phase 3 study. Lancet Oncol. 2016;17(2):200-11.
13. Byrd JC, Brown JR, O'Brien S, Barrientos JC, Kay NE, Reddy NM, et al. Ibrutinib versus ofatumumab in previously treated chronic lymphoid leukemia. N Engl J Med. 2014;371(3):213-23.
14. Wierda WG, Allan JN, Siddiqi T, Kipps TJ, Opat S, Tedeschi A, et al. Ibrutinib Plus Venetoclax for First-Line Treatment of Chronic Lymphocytic Leukemia: Primary Analysis Results From the Minimal Residual Disease Cohort of the Randomized Phase II CAPTIVATE Study. J Clin Oncol. 2021;39(34):3853-65.

15. Furman RR, Sharman JP, Coutre SE, Cheson BD, Pagel JM, Hillmen P, et al. Idelalisib and rituximab in relapsed chronic lymphocytic leukemia. N Engl J Med. 2014;370(11):997-1007.
16. Jones JA, Robak T, Brown JR, Awan FT, Badoux X, Coutre S, et al. Efficacy and safety of idelalisib in combination with ofatumumab for previously treated chronic lymphocytic leukaemia: an open-label, randomised phase 3 trial. Lancet Haematol. 2017;4(3):e114-e26.
17. Zelenetz AD, Barrientos JC, Brown JR, Coiffier B, Delgado J, Egyed M, et al. Idelalisib or placebo in combination with bendamustine and rituximab in patients with relapsed or refractory chronic lymphocytic leukaemia: interim results from a phase 3, randomised, double-blind, placebo-controlled trial. Lancet Oncol. 2017;18(3):297-311.
18. Fink AM, Bahlo J, Robrecht S, Al-Sawaf O, Aldaoud A, Hebart H, et al. Lenalidomide maintenance after first-line therapy for high-risk chronic lymphocytic leukaemia (CLLM1): final results from a randomised, double-blind, phase 3 study. Lancet Haematol. 2017;4(10):e475-e86.
19. Chanan-Khan AA, Zaritskey A, Egyed M, Vokurka S, Semochkin S, Schuh A, et al. Lenalidomide maintenance therapy in previously treated chronic lymphocytic leukaemia (CONTINUUM): a randomised, double-blind, placebo-controlled, phase 3 trial. Lancet Haematol. 2017;4(11):e534-e43.
20. Jindal N, Lad DP, Malhotra P, Prakash G, Khadwal A, Jain A, et al. Randomized controlled trial of individualized, low dose, fixed duration lenalidomide maintenance versus observation after frontline chemo-immunotherapy in CLL. Leuk Lymphoma. 2021;62(7):1674-81.
21. Goede V, Fischer K, Busch R, Engelke A, Eichhorst B, Wendtner CM, et al. Obinutuzumab plus chlorambucil in patients with CLL and coexisting conditions. N Engl J Med. 2014;370(12):1101-10.
22. Hillmen P, Robak T, Janssens A, Babu KG, Kloczko J, Grosicki S, et al. Chlorambucil plus ofatumumab versus chlorambucil alone in previously untreated patients with chronic lymphocytic leukaemia (COMPLEMENT 1): a randomised, multicentre, open-label phase 3 trial. Lancet. 2015;385(9980):1873-83.
23. Robak T, Warzocha K, Govind Babu K, Kulyaba Y, Kuliczkowski K, Abdulkadyrov K, et al. Ofatumumab plus fludarabine and cyclophosphamide in relapsed chronic lymphocytic leukemia: results from the COMPLEMENT 2 trial. Leuk Lymphoma. 2017;58(5):1084-93.
24. van Oers MH, Kuliczkowski K, Smolej L, Petrini M, Offner F, Grosicki S, et al. Ofatumumab maintenance versus observation in relapsed chronic lymphocytic leukaemia (PROLONG): an open-label, multicentre, randomised phase 3 study. Lancet Oncol. 2015;16(13):1370-9.
25. Hallek M, Fischer K, Fingerle-Rowson G, Fink AM, Busch R, Mayer J, et al. Addition of rituximab to fludarabine and cyclophosphamide in patients with chronic lymphocytic leukaemia: a randomised, open-label, phase 3 trial. Lancet. 2010;376(9747):1164-74.
26. Robak T, Dmoszynska A, Solal-Céligny P, Warzocha K, Loscertales J, Catalano J, et al. Rituximab plus fludarabine and cyclophosphamide prolongs progression-free survival compared with fludarabine and cyclophosphamide alone in previously treated chronic lymphocytic leukemia. J Clin Oncol. 2010;28(10):1756-65.
27. Greil R, Obrtlíková P, Smolej L, Kozák T, Steurer M, Andel J, et al. Rituximab maintenance versus observation alone in patients with chronic lymphocytic leukaemia who respond to first-line or second-line rituximab-containing chemoimmunotherapy: final results of the AGMT CLL-8a Mabtenance randomised trial. Lancet Haematol. 2016;3(7):e317-29.
28. Eichhorst B, Fink AM, Bahlo J, Busch R, Kovacs G, Maurer C, et al. First-line chemoimmunotherapy with bendamustine and rituximab versus fludarabine, cyclophosphamide, and rituximab in patients with advanced chronic lymphocytic leukaemia (CLL10): an international, open-label, randomised, phase 3, non-inferiority trial. Lancet Oncol. 2016;17(7):928-42.

29. Dartigeas C, Van Den Neste E, Léger J, Maisonneuve H, Berthou C, Dilhuydy MS, et al. Rituximab maintenance versus observation following abbreviated induction with chemoimmunotherapy in elderly patients with previously untreated chronic lymphocytic leukaemia (CLL 2007 SA): an open-label, randomised phase 3 study. Lancet Haematol. 2018;5(2):e82-e94.
30. Fischer K, Al-Sawaf O, Bahlo J, Fink AM, Tandon M, Dixon M, et al. Venetoclax and Obinutuzumab in Patients with CLL and Coexisting Conditions. N Engl J Med. 2019;380(23):2225-36.
31. Seymour JF, Kipps TJ, Eichhorst B, Hillmen P, D'Rozario J, Assouline S, et al. Venetoclax-Rituximab in Relapsed or Refractory Chronic Lymphocytic Leukemia. N Engl J Med. 2018;378(12):1107-20.
32. Hillmen P, Skotnicki AB, Robak T, Jaksic B, Dmoszynska A, Wu J, et al. Alemtuzumab compared with chlorambucil as first-line therapy for chronic lymphocytic leukemia. J Clin Oncol. 2007;25(35):5616-23.
33. Huang X, Qiu L, Jin J, Zhou D, Chen X, Hou M, et al. Ibrutinib versus rituximab in relapsed or refractory chronic lymphocytic leukemia or small lymphocytic lymphoma: a randomized, open-label phase 3 study. Cancer Med. 2018;7(4):1043-55.
34. Burger JA, Tedeschi A, Barr PM, Robak T, Owen C, Ghia P, et al. Ibrutinib as Initial Therapy for Patients with Chronic Lymphocytic Leukemia. N Engl J Med. 2015;373(25):2425-37.
35. Moreno C, Greil R, Demirkan F, Tedeschi A, Anz B, Larratt L, et al. Ibrutinib plus obinutuzumab versus chlorambucil plus obinutuzumab in first-line treatment of chronic lymphocytic leukaemia (iLLUMINATE): a multicentre, randomised, open-label, phase 3 trial. Lancet Oncol. 2019;20(1):43-56.
36. Foà R, Del Giudice I, Cuneo A, Del Poeta G, Ciolli S, Di Raimondo F, et al. Chlorambucil plus rituximab with or without maintenance rituximab as first-line treatment for elderly chronic lymphocytic leukemia patients. Am J Hematol. 2014;89(5):480-6.
37. Hillmen P, Cohen DR, Cocks K, Pettitt A, Sayala HA, Rawstron AC, et al. A randomized phase II trial of fludarabine, cyclophosphamide and mitoxantrone (FCM) with or without rituximab in previously treated chronic lymphocytic leukaemia. Br J Haematol. 2011;152(5):570-8.
38. Sharman JP, Brander DM, Mato AR, Ghosh N, Schuster SJ, Kambhampati S, et al. Ublituximab plus ibrutinib versus ibrutinib alone for patients with relapsed or refractory high-risk chronic lymphocytic leukaemia (GENUINE): a phase 3, multicentre, open-label, randomised trial. Lancet Haematol. 2021;8(4):e254-e66.
39. Österborg A, Udvardy M, Zaritskey A, Andersson PO, Grosicki S, Mazur G, et al. Phase III, randomized study of ofatumumab versus physicians' choice of therapy and standard versus extended-length ofatumumab in patients with bulky fludarabine-refractory chronic lymphocytic leukemia. Leuk Lymphoma. 2016;57(9):2037-46.
40. Flinn IW, Hillmen P, Montillo M, Nagy Z, Illés Á, Etienne G, et al. The phase 3 DUO trial: duvelisib vs ofatumumab in relapsed and refractory CLL/SLL. Blood. 2018;132(23):2446-55.
